# Nano-Integrated Suspended Polymeric Microfluidics (SPMF) Platform for Ultra-Sensitive Bio-Molecular Recognition of Bovine Growth Hormones

**DOI:** 10.1038/s41598-017-11300-2

**Published:** 2017-09-08

**Authors:** Hamid Sadabadi, Muthukumaran Packirisamy

**Affiliations:** 1 Optical Bio-Micro Systems Laboratory, Department of Mechanical Engineering, Concordia University, EV 4.139, 1455 de Maisonneuve Blvd. W, Montreal, QC H3G 1M8 Canada; 2 BioMEMS and Bioinspired Microfluidic Laboratory, Department of Mechanical Engineering, University of Calgary, 2500 University Dr. N.W., EEEL 455B, Calgary, AB T2N 1N4 Canada

## Abstract

The development of sensitive platforms for the detection of biomolecules recognition is an extremely important problem in clinical diagnostics. In microcantilever (MC) transducers, surface-stress is induced upon bimolecular interaction which is translated into MC deflection. This paper presents a cost-effective and ultra-sensitive MC-based biosensing platform. To address these goals, the need for costly high-resolution read-out system has been eliminated by reducing the cantilever compliance through developing a polymer-based cantilever. Furthermore a microfluidic system has been integrated with the MC in order to enhance sensitivity and response time and to reduce analytes consumption. Gold nanoparticles (AuNPs) are synthesized on the surface of suspended microfluidics as the selective layer for biomolecule immobilization. The biosensing results show significant improvement in the sensitivity of the proposed platform compared with available silicon MC biosensor. A detection limit of 2 ng/ml (100pM) is obtained for the detection of bovine growth hormones. The results validated successful application of suspended polymeric microfluidics (SPMF) as the next generation of biosensing platforms which could enable femtomolar (fM) biomolecular recognition detection.

## Introduction

Advances in micro-nanofabrication techniques are enabling a wide range of new technologies. Progresses in the fabrication of transducer technologies with more comprehensive understanding of the governing rules of biomolecular interactions, have led to rapid advancements in biosensors developments^1–3^.

MC biosensors provide large deflection for exquisite mass resolution^3, 4^. The compliance of the mechanical sensors related to their stiffness can be tuned in the order of three by scaling the device dimensions^5^. The mechanical response is generally the deflection of the cantilever that can be measured using different methods including optical, capacitive, and electrical. Upon the recognition of the biomolecules on the cantilever’s transduction surface coated with selective layer, a surface stress is induced which results in cantilever bending. The magnitude of bending depends on the analytes concentrations^6–10^.

MC sensors operate in two modes: the *static mode* in which deflection is sensitive to surface stress changes and the *dynamic mode* in which resonance frequency shift is sensitive to mass variations^2, 11^.

In addition to the pioneering of MC sensors^2, 12, 13^, one of the first successful examples on MC biosensing has been proposed by Fritz *et al*.^4^ for label-free DNA hybridization detection and reached a limit of detection of 80 nM for the complementary DNA oligonucleotide. The resulting beam deflection of 6-10 nm was measured through optical methods. MCs have wide applications in biosensing such as circulating tumor cells (CTCs) detection^14^, virus detection^15, 16^, DNA hybridization^17–19^, PSA detection^20, 21^ and Ag/Ab interaction detection^22^.

Microfluidic systems have several advantages once integrated with biosensing platforms. Mainly they reduce sample volumes and target analyte, fast response times, high throughput, enhanced analytical performance, less waste, low unit cost, reduced energy consumption, and reduced dimensions when compared to macro-scale techniques^4^.

Burg^23^ proposed microchannels integrated MC referred as SMR (suspended microchannel resonator) with applications in mass detection^24–30^. Such a platform is capable of achieving resolutions down to femto grams^31^ or even atto grams^32^.

One of the drawbacks of MC biosensors is the high amount of analytes required for experiments as the cantilevers need to be submerged in the analytes. In addition, the cantilever bending read-out system should be sensitive enough to measure deflections in the range of few nanometers^4, 33, 34^. Main applications of microfluidic integrated MCs (SMRs) are limited to mass detection. This is because since all reported SMRs are made of silicon, the typical anticipated deflection of a SMR for biosensing even with high concentrations of analyte will lie in the range of a few pm (≈50 pm) which are far beyond the limits of current measuring methods^4^. Furthermore, the manufacturing of SMR is complicated and encompasses multiple cumbersome fabrication steps. A recently published review paper [1] explains the sensitivity and response time of different sensing mechanism of biosensors. They show that for example SPR sensors generally have a very good response time (few seconds), while their detection limit is generally high. In contrast, in MCs, their detection limit they can achieve is very promising while their response time is in the range of even few hours. In order to build a sensitive platform with quick response time, one method is to take advantage of multiple sensing mechanisms in a single platform.

To address these issues, we propose a low-cost PDMS (Polydimethylsiloxane) microcantilever with integrated microfluidics biosensor for protein-protein interaction detection. To create a selective layer, AuNPs are synthesized and immobilized onto the microfluidic channel in the form of a nanocomposite films by using an *in-situ* method which has been fully investigated herein^35, 36^. In the last decade, nanometal-polymer composite in particular gold-PDMS nanocomposite films attracted many researches for biosensing applications due to their enhanced optical properties associated with localized surface plasmon resonance (LSPR)^37, 38^.

Due to the high flexibility of the PDMS with a Young’s modulus that is five orders of magnitude less compared to silicon, the resulting deflection from the induced surface stress in a PDMS MC can be augmented in the same order. Hence the sensitivity and limit of detection are considerably enhanced in a PDMS cantilever compared to a silicon cantilever.

## Results and Discussions

### Fabrication of SPMF sensing platform

**Figure 1:**
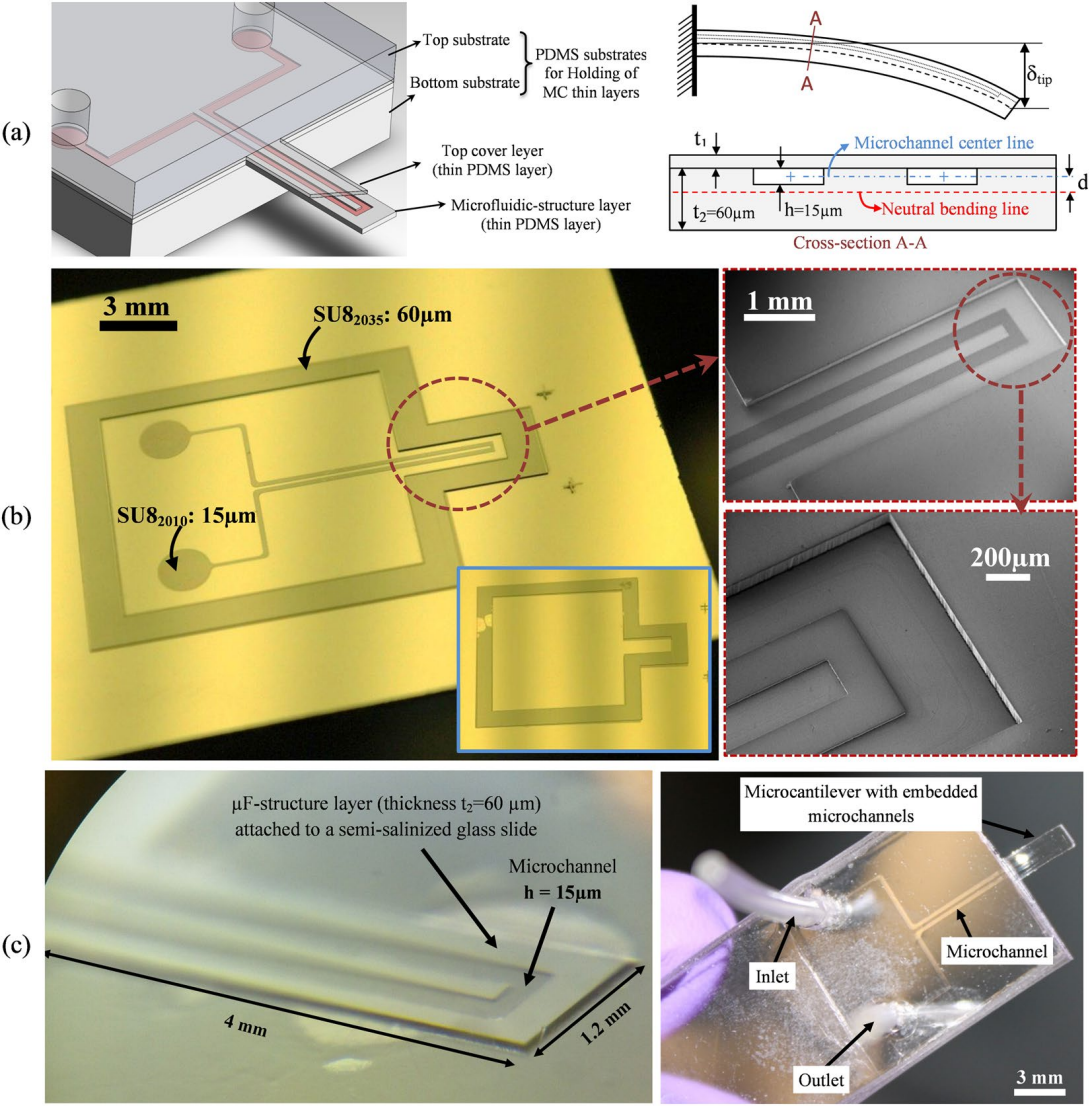
Design and fabrication of PDMS cantilever with buried microchannel; (**a**) schematic of the cantilever, its layers and the cross-section of the cantilever. The parameters that used to design and optimize the cantilever are shown; (**b**) images of the SU8 mold for fabricating of thin layers. The SU-8 mold for bottom µF-structure thin layer is shown and its SEM images demonstrate two different SU-8 levels. The inset shows the SU-8 mold of top thin layer; (**c**) fabricated µF-structure layer with the buried microchannel with the depth of 15 µm on a semi-salinized glass slide. Fabricated SPMF platform. The thickness of cantilever is 165 µm.

A PDMS microcantilever with integrated microfluidics has been fabricated as the sensing platform. For the fabrication of SPMF, SU-8 photoresist patterning procedure was used to prepare two molds which were used further to make different layers of the MC. A schematic of the SPMF and its cross-section are shown in Fig. 1-a. The cantilever is made of two thin layers and the supporting substrates are made of two thick layers. These layers from top to bottom include: (1) top substrate layer for holding of the cantilever; (2) top cover layer to close the channel with the thickness of *t*_1_; (3) a microfluidic (µF)-structure layer with the thickness of *t*_2_ containing the buried microchannel of height *h* (4) bottom substrate layer for holding of the cantilever.

The SU-8 mold used for the fabrication of microfluidic-structure layer contains two SU-8 layers with thicknesses of 15 µm and 60 µm. A layer with thickness of *h* = 15 µm forms the buried microchannel and the other layer forms the microfluidic-structure layer.

Images of the fabricated mold for the µF-structure layer of SPMF are shown in Fig. 1-b. In this figure, the close-up images show two SU-8 layers. The inset image shows the mold that was used for the fabrication of thin cover layer. The fabrication procedures of both molds are explained in the supplementary information. Figure 1-c shows a µF-structure thin layer with microchannel features on it on a semi-salinized glass slide. This figure also shows the final SPMF platform after bonding of all four layers. The thicknesses of the cover layer and µF-structure layer were selected as t_1_ = 105 µm and t_2_ = 60 µm which make the MC thickness as 165 µm. Since the induced surface-stress results in bending of the cantilever, the distance between microchannel center line and neutral axis of the cantilever, variable *d* in Fig. 1-a, is critical and needs to be optimized for maximum deflection of the cantilever to improve the sensitivity of the sensor. The process of MC design optimization for biosensing application is explained in detail in supplementary information.

### Design of Cantilever

A schematic of a microcantilever and its cross-section are shown in Fig. 1-a. Upon interaction of immobilized biomolecules in the buried microfluidic channel, a surface-stress will be induced that bends the cantilever.

Unbalanced stresses relative to the neutral axis of the cantilever cross-section create a bending moment with respect to the neutral axis which makes the cantilever to deflect. In the proposed microcantilever, the surface-stress is assumed to be induced inside the microchannels. Therefore, the thickness of the cantilever cover layer, parameter *t*_1_, plays an important role in the location of the neutral axis. Indeed, changing the thickness of top layer can result in the displacement of bending neutral line. For a known induced surface-stress, variable *d*, the distance between the neutral line and the microchannel center line, has a direct impact on the cantilever bending. The variable *d*, can be obtained from the geometry of the cross-section, and can be calculated mathematically by Equation (1):

1 $$\{\begin{array}{llllll}d & = & (\frac{{t}_{1}+{t}_{2}}{2})-({t}_{1}+\frac{h}{2}) & {t}_{1} &  <  & {t}_{2}-h\\ d & = & 0 & {t}_{1} & = & {t}_{2}-h\\ d & = & ({t}_{1}+\frac{h}{2})-(\frac{{t}_{1}+{t}_{2}}{2}) & {t}_{1} &  >  & {t}_{2}-h\end{array}$$

**Figure 2:**
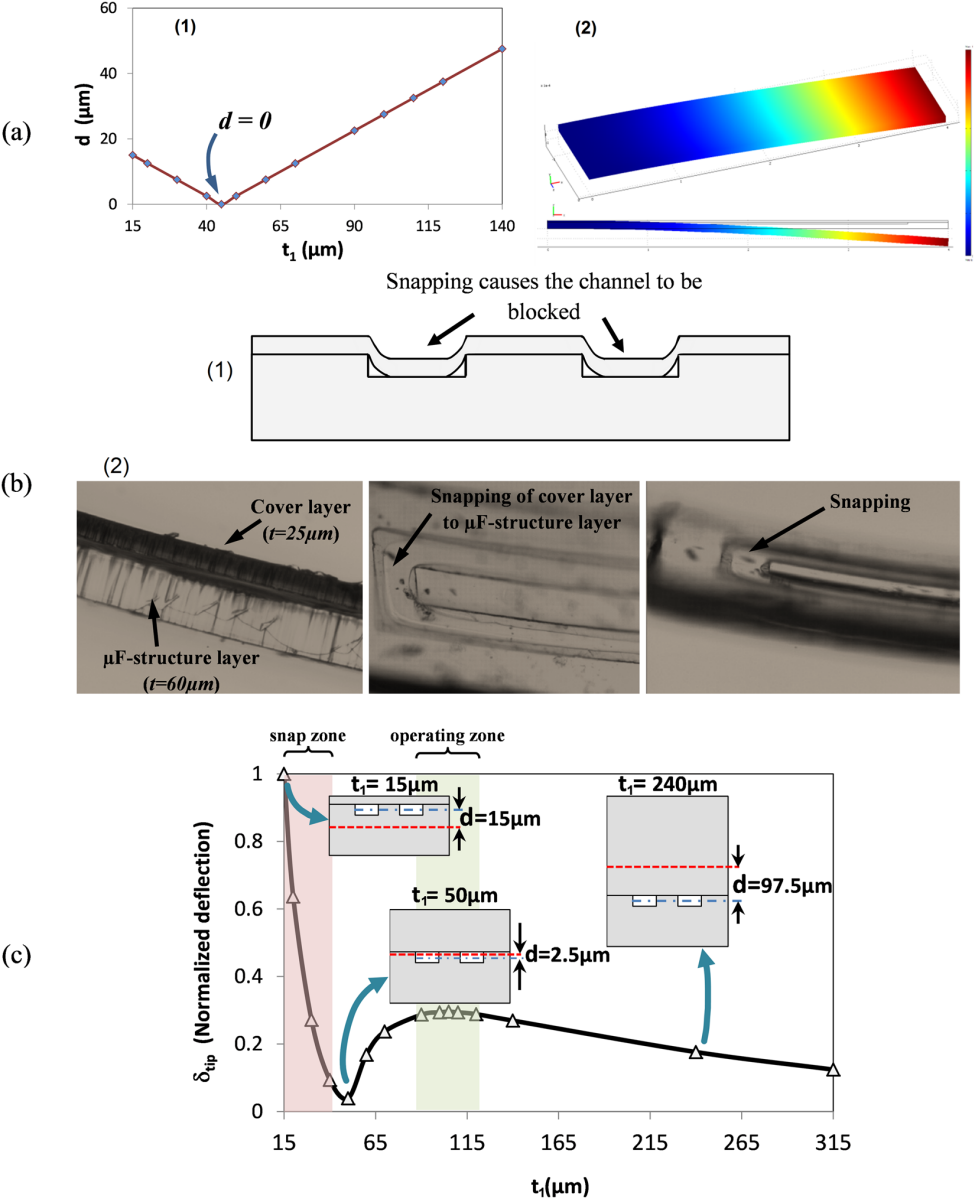
Design and optimization of SPMF: (**a**) variation of variable *d* with respect to the thickness of cantilever cover layer *t*_*1*_ (left); FE simulation of a PDMS suspended microfluidics deflection under the influence of surface-stress at microchannel (right); (**b**) schematics of closure of the microchannel due to snapping problem of two thin layers due to high interaction between the plasma activated surfaces (1), fabricated SPMF with *t*_*1*_ = *25* *μm* while two layers are snapped (2); (**c**) variation of cantilever tip deflection with respect to cantilever top layer thickness, *t*_*1*_, for the optimization of the SPMF thickness for biosensing application. The optimized thickness has been selected for the range (90 µm < t_1_ < 120 µm) which results in the highest tip deflection while the snapping occurrence is minimized.

Where *t*_1_ is the thickness of cover layer, *t*_2_ = 60 µm is the thickness of µF-structure layer, and *h* = 15 µm is the depth of the buried microchannel. The variation of *d* with respect to *t*_1_ is shown in Fig. 2-(a-1).

As seen in this graph, when *t*_1_ = 45 µm, the value of *d* = *0*, which means the center of microchannel is placed at the cantilever neutral axis. In this condition, the induced surface-stress can only elongate the cantilever with no bending (*δ*_*tip*_ = *0*) which is not suitable for biosensing application. Upon increasing *t*_1_, first *d* decreases linearly and reaches to *d* = *0* (at *t*_1_ = *45* *µm*), then it increases linearly as a function of *t*_1_.One can select the variable *d* as maximum as possible to maximize the deflection. In contrary, the increase of *d*, increase the stiffness of the cantilever which reduces the cantilever bending for a given surface-stress. Hence an optimization needs to be done with respect to variables *d* and *t*_1_ to maximize the tip deflection of the cantilever, *δ*_*tip*_.

#### Cantilever Optimization for biosensing applications

To find the optimum value of *t*_1_, the deflection of cantilever under a known induced surface-stress was modeled. The COMSOL® Multiphysics MEMS module was used for the finite element (FE) analysis of cantilever using Euler-Bernoulli equation. A 3D CAD model is used for the simulation. The whole 3D volume is discretized with the maximum element size of 10 µm. A convergence study has been done to obtain suitable mesh size.

PDMS can be manufactured with different ratios of base to curing agent which alter its mechanical properties significantly. A ratio of 10:1 is used in this study. It should be noted that not only PDMS with different ratios, but also 10:1 ratio PDMS with different thicknesses exhibit different elasticity. In another word, it has been shown that both the mechanical strength and the Young’s modulus of the PDMS membranes are thickness dependent. In particular, for the PDMS membrane with a thickness of less than 200 µm, there is a high non-linearity relation between the mechanical properties and thickness of the membrane. There are few published literatures that discussed this relation in detail. We have used the data from one of these previously published works to perform the finite element simulation. The thickness dependent mechanical properties of PDMS have been used in the FE model to simulate the MC with different thicknesses^39^.

The mechanical properties used for the simulation are ρ = 0.97 g/cm^3^ and ν = 0.5 (Poisson ratio). Since there is a linear relationship between the *δ*_*tip*_ and surface-stress *σ* for each geometrical embodiment of the cantilever, the magnitude of the surface-stress is assumed as unit (*Nm* ^*−*1^) for simplicity. The simulated cantilever with the real dimensions and its deflection is shown in Fig. 2-(a-2). In this figure the deflection of cantilever is normalized while the blue color shows zero deflection, the red color corresponds to the maximum deflection of 1.

The cantilever with different top layer thicknesses was simulated. For each case the value of Young’s modulus of the PDMS was selected based on the thickness of the cantilever from a published literature^39^. The normalized tip deflection *δ*_*tip*_ against the thickness *t*_1_ was plotted in Fig. 2-c. From this plot it can be seen that for a thin top layer with the thickness of *t*_1_ = 1*5* *µm*, we have the maximum deflection. By increasing the thickness, the deflection will be decreased as a result of reducing of the parameter *d*. By approaching to *d* = 0, the deflection will approach to zero. Upon further increasing of *t*_1_, the deflection will first increase and then will decrease. The increase is due to increasing of parameter *d*, however, by keep increasing of the thickness, the stiffness of the cantilever will increase which results in decreasing of the cantilever deflection for a given surface-stress. Therefore, from this figure, it can be inferred that the optimized thickness of the top layer can be either in the range of *t*_1_ = 1*5*–*25* *µm* for absolute maximum deflection or in the range of *t*_1_ = *90*–1*20* *µm* for a local maximum.

#### Snapping problem

During the fabrication of the SPMF, some manufacturing limitations exist which restricts the thickness range of thin layer that can be chosen for the cantilever. One of the last steps in the fabrication procedure of SPMF is the plasma bonding of thin layers. Our results show that when the thickness of the cover layer *t*_1_ is very low, upon its bonding with the µF-structure layer, the channels in the cantilever will be blocked due to snapping of the top layer towards the bottom layer. The closure of the channel happened due to very high tension of plasma activated PDMS surfaces from both sides which cause the surfaces to stick and then bond to each other. It results in blockage of the microfluidic channel. This phenomenon is shown schematically in Fig. 2-(b-1) and a real snapping occurrence is shown in Fig. 2-(b-2).

#### Optimized cantilever dimensions

As discussed earlier, for the fabrication of thin cover layer, molds with five (5) different cover layers were fabricated for testing. Our fabrication results show that the cover layer with the thicknesses less than *t*_1_ = *40* *µm* will result in the snapping of the channel. Therefore it can be concluded that the thickness of cover layer should be selected higher than *40* *µm* (*t*_1_ > *40* *µm)*in order to avoid the snapping problem.

It should be noted that the mentioned threshold value of *t*_1_ = *40* *µm* to avoid snapping, is strongly dependent on the microchannel width and it will decrease when the channel width is reduced. As a result of the snapping problem, the optimized thickness range of *t*_1_ = *15–25* *µm* cannot be selected for the fabrication of microcantilever. Hence, the only possible range for the fabrication of MC is *90* *µm* < *t*_*1*_ < *120* *µm* and optimum value of *t*_*1*_ = 105 µm would results in maximum cantilever deflection. The fabricated cantilever with optimum dimensions has a length of 4 mm, width of 1.2 mm and thickness of 165 µm. the width of the buried channel is selected to be 200 µm with the depth of 15 µm.

### Integration of gold nanoparticles (AuNPs) into microchannel

An *in-situ* synthesis method that was previously developed^36^ were used to integrate AuNPs into the PDMS network in the buried microchannel of MC. For this purpose, a 2% aqueous solution of chloroauric acid was introduced into the microfluidic channel. Then the chip was kept at room temperature for 48 hrs. After the incubation time, the channel turns to red showing the presence of AuNPs inside the microchannel

**Figure 3:**
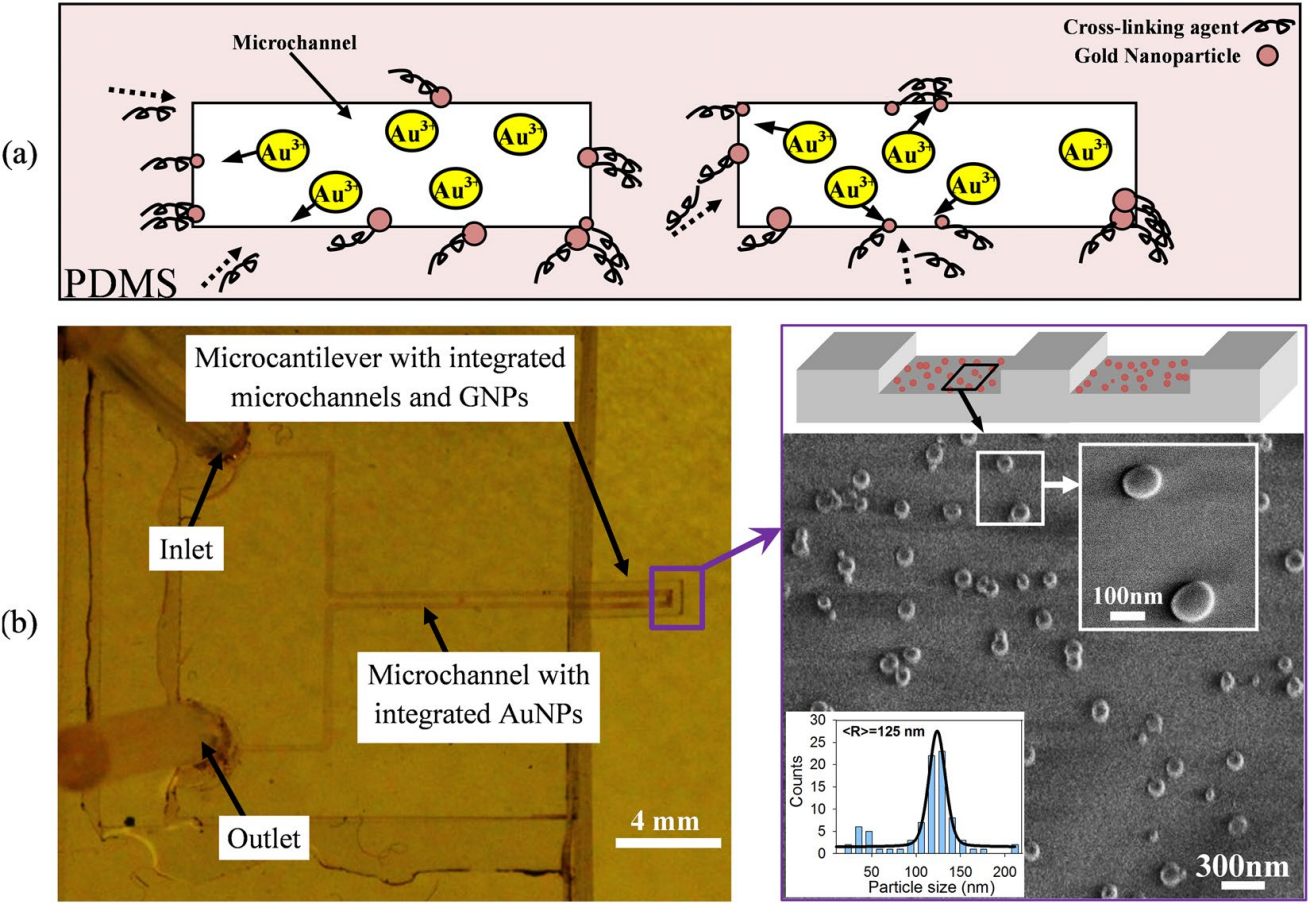
AuNPs are integrated onto the surface of PDMS in the buried microchannels of SPMF to form an Au-PDMS nanocomposite film as the selective layer for biomolecule immobilization: (**a**) illustration of the *in-situ* reaction at the cross-section of microcantilever and the migration of reducing agent towards the channel wall; (**b**) the SEM image shows the shapes and size distribution of AuNPs inside the channel. The plot shows the particles size distribution is within the range of 125 ± 5 nm.

The curing (cross-linking) agent in PDMS compound reduces the gold ions to gold nanoparticles embedded into the polymer network^35^. A schematic of the process of *in-situ* reaction at the cross-section of microcantilever and formation of AuNPs are illustrated in Fig. 3-a. After *in-situ* synthesis completion, heat treatment was used in order to improve the size distribution of AuNPs in PDMS^35^. Annealing process was done by keeping SPMF at 300 °C for 10 min, to reduce the particles aggregation and to create a more uniform distribution of particles on PDMS surface. SEM images of the AuNPs are obtained from the channel show uniform particle size with average size of 125 ± 5 nm as shown in Fig. 3-b. The synthesis method is explained in supplementary information.

### Biosensing

Label-free biosensing tests of bovine growth hormone (BGH) have been performed by using the annealed SPMF. The BGH antibodies (anti-BGH) in mouse and its corresponding antigens (Ag) were used for biosensing. The biosensing protocol is explained in supplementary information.

**Figure 4:**
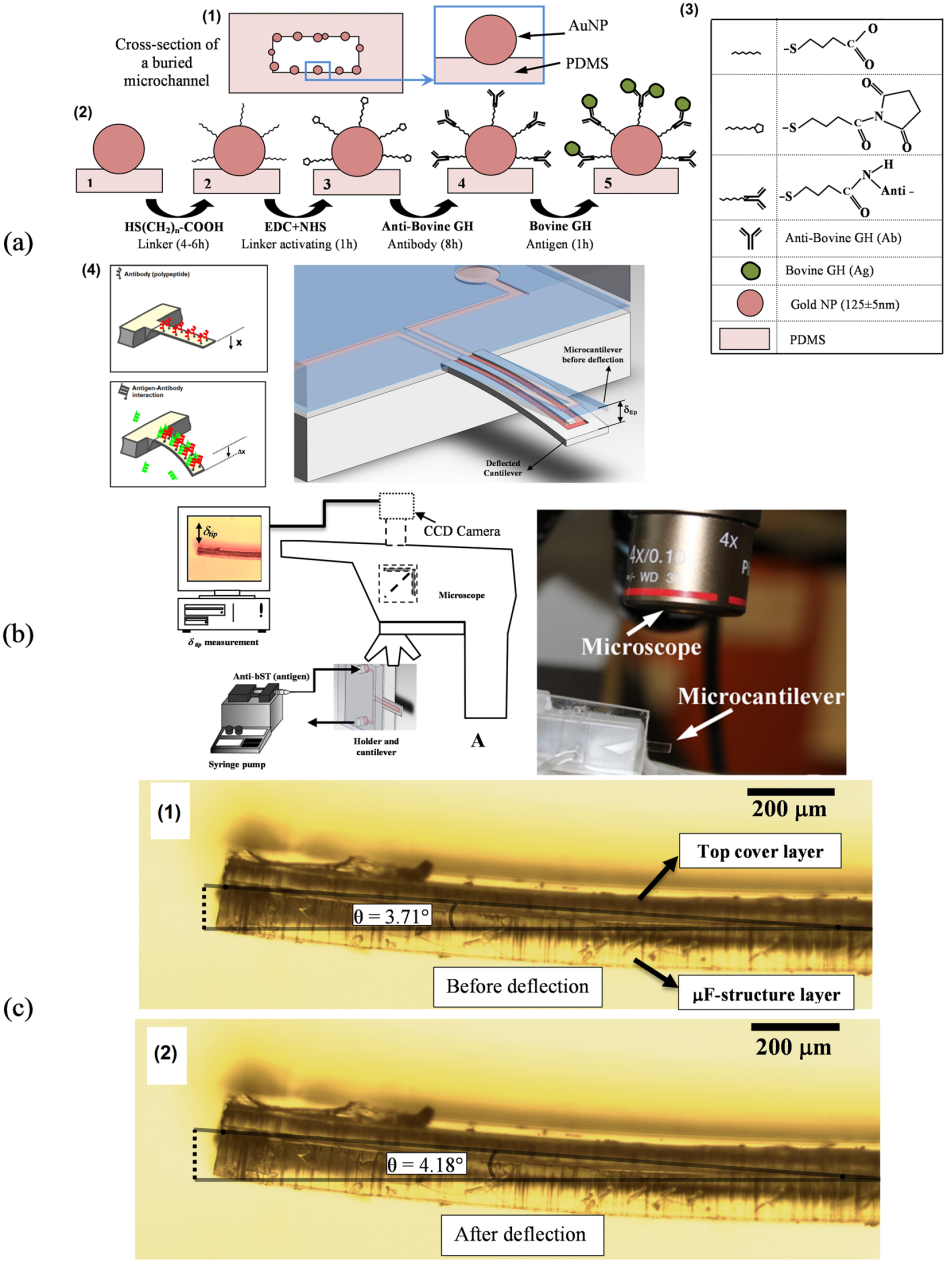
Test set-up for the detection of bovine growth hormone by using the proposed SPMF platform: (**a**) schematics of AuNPs on the surface of buried microchannel and the biosensing protocol to immobilize Ab and Ag on the surface of AuNPs. The cantilever was bent due to the induced surface-stress from Ag–Ab interaction; (**b**) schematic of cantilever deflection measurement setup and the device under test. A CCD camera track the cantilever tip though an image processing module during the biosensing test; (**c**) MC bending defined by the deflection measured from the bending of the cantilever along its length due to surface stress and not rotating at the base of the cantilever.

The schematic of formation of AuNPs on the surface of buried microchannel of SPMF is shown in Fig. 4-(a-1). The immobilization steps of antibodies (Ab) on the surface of AuNPs are schematically shown in Fig. 4-(a-2,a-3). Figures 4-(a-4) shows that the cantilever response in form of bending due to the induced surface-stress from Ag–Ab interaction in the last step of biosensing protocol.

The magnitude of cantilever deflection is proportional to the induced surface stress which is related to the concentration of Ag in solution. The schematic of cantilever deflection measurement setup and the device under test (DUT) are shown in Fig. 4-b. The biosensing experiments were performed under an optical microscope that tracks the cantilever tip though an image processing module that measures SPMF tip deflection in real time. Different antigen concentrations were tested to obtain the detection limit of the proposed integrated cantilever biosensor. It should be noted that the cantilever bends down (and not simply rotates) due to the induced surface-stress along the buried microchannel surface of the cantilever. In order to show this fact, two fixed points were assumed along the cantilever and then a line was passed through them to measure the cantilever angle before and after deflection as shown in Fig. 4-(c-1 and c-2). Variation in the slope of this line proves that the cantilever is bent along its length and not simply moved or hinged about its base. It is assumed that the base of the cantilever is fixed and rigid.

The biosensing experiments were performed using a SPMF with dimensions of 4 mm long, 1.2 mm width and 165 µm thick. Anti-BGH was injected into the buried microchannel at the last step of biosensing and at the same time the deflection of the cantilever was recorded using a CCD camera that was mounted on an optical microscope.

**Figure 5:**
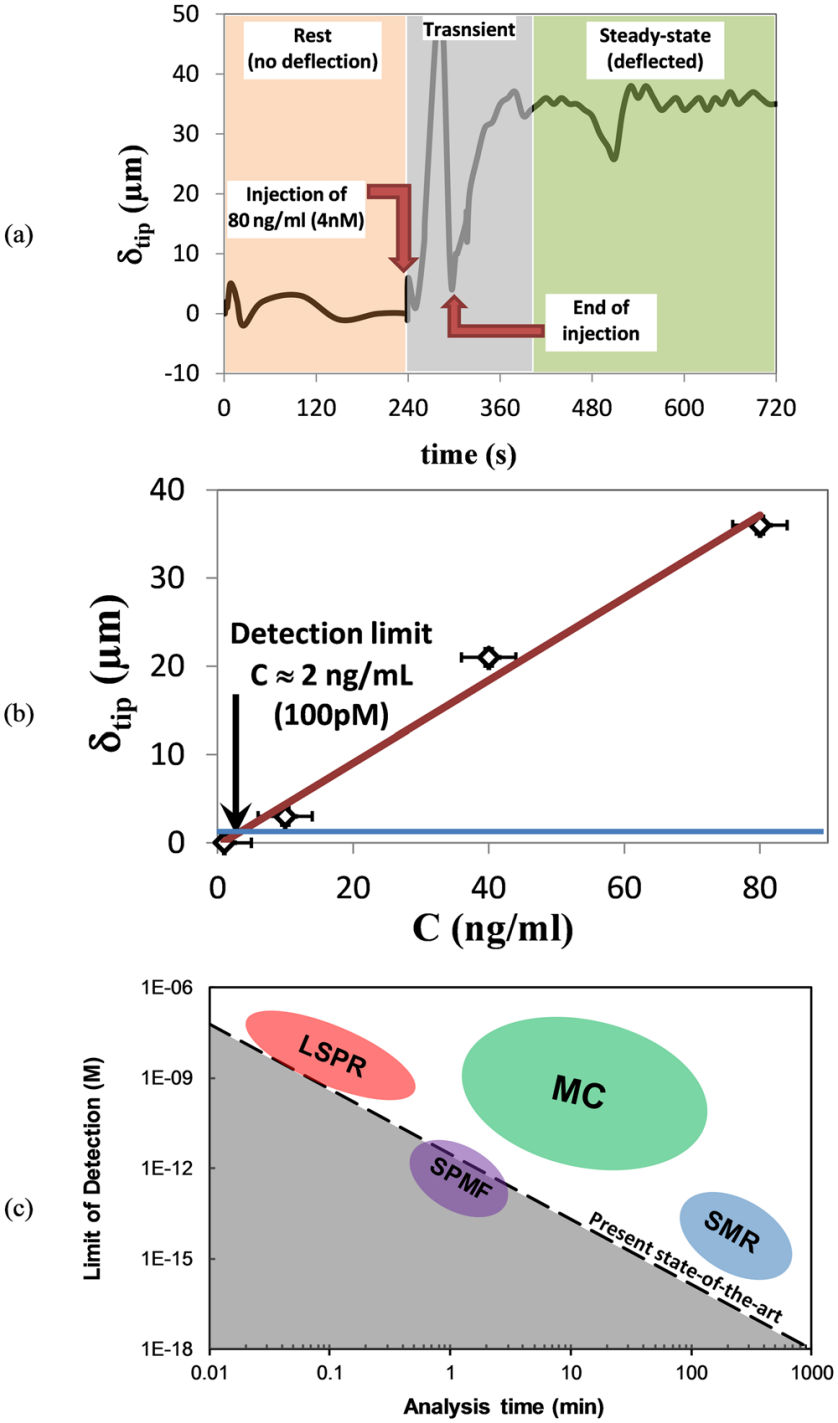
Using of SPMF for Ag-Ab interaction detection of BGH in mouse: (**a**) response of the cantilever when 4 nM anti-BGH is injected into the SPMF. Three phases of biosensing includes: (i) before injection of Ag; (ii) during the stress induction; and (iii) the final response. A mean steady-state deflection of δ_tip_ = 35 µm was observed within 90 sec from the end of Ag injection; (**b**) mean steady-state deflection of the cantilever tip due to different antigen concentration and calculation of detection limit. A limit of detection of 2 ng/ml (100 pm) was observed with a minimum deflection resolution of 1 µm; (**c**) the results of the presented SPMF showing a big improvement in the analysis time and limit of detection of current state-of-the-art biosensors. Further improvement of cantilever readout system can promote the detection limit of SMPF even into untouched femto-molar and even atto-molar region [Adapted from J. Arlett *et al*.^1^].

Figure 5-a shows the response of the cantilever deflection for the duration of 12 min during the last step of a biosensing experiment upon injection of 4 nM concentration of growth hormones (Antigens, Ag). The antigen solution was injected into the microchannel at time *t* = *240* *sec* with a flow rate of 10 µl/min. Then the syringe-pump was stopped at *t* = *310* *sec* when the antigen solution filled the buried microfluidic completely meaning that the antigens are in contact with immobilized antibodies on the AuNPs surface. Different time zones can be observed during the biosensing procedure corresponding to different phases that occur during the biomolecular interaction as marked in Fig. 5-a. The response graph shows that the cantilever is deflected with a mean steady-state deflection of *δ*_*tip*_ = *35* *µm* (mean deflection value in the steady-state response section) from the rest position as the result of induced surface–stress due to Ag-Ab interaction. In order to compensate for the weight of the fluid in the channel, the cantilever initial rest position was measured in all of the tests when the channels were full with PBS prior to testing.

Figure 5-a also illustrates that the response of cantilever encompasses three phases during the interaction. The first phase is when the cantilever is at its rest condition with the deflection of δ_tip_ = 0. The second phase starts at t = 240 sec when the syringe pump starts to inject the Ag solution. This phase can be considered as a transient phase and it includes disturbances and noises and it lasts for around 160 sec when the cantilever tip reached to its final steady-state response.

The deflection of the cantilever tip due to different antigen concentration has been measured in order to calculate the limit of detection of the sensing platform. The biosensing results for four different concentration are presented in Fig. 5-b. Assuming a linear relation between the cantilever deflection and the concentration of the antigen, the limit of detection of the biosensing platform is obtained to be 2 ng/ml (100 pM) for a minimum deflection resolution of 1 µm.

Another promising achievement from results is the biosensing response time of the cantilever due to the induced surface-stress. As it can be seen from the Fig. 5-a, the cantilever has reached its steady-state position within 90 sec after the injection of Ag solution. The fast response time can be accounted by the occurrence of Ag-Ab interaction in a microfluidic environment. For different Ag concentrations, the response time of the cantilever towards steady-state deflection was 80 ± 40 sec showing a very fast response time compared with MC-based sensing platforms with few hours settling time.

In microfluidic-based sensing systems, due to the confinement effect, an improvement in the response time is generally observed compared to conventional sensing systems. Indeed, in biosensing with Si-cantilevers, due to the diffusion-based sensing of the submerged cantilever, the sensing mechanism is generally slow (in the order of few minutes). In the presented platform, the Ag solution pass over the immobilized Ab on the channel and due to direct contact of Ag/Ab, the time response has been improved. Other possible reasons for the improved response time is the integration of nano islands on the microfluidics surfaces that enhances the bio-interactions due to higher surface area which increased binding sites leading better sensitivity and response time.

As seen in Fig. 5-c, SPMF provides great promise to cross the limitation of the present state-of-the-art sensing techniques including microcantilevers and would eventually lead to ultra-sensitive detection at femto and even atto molar concentrations.

## Conclusions

To the best of our knowledge, for the first time, a biosensing platform made of PDMS microcantilever with buried microchannel was proposed. AuNPs are integrated into the suspended-microfluidics using an *in-situ* method to provide a suitable selective layer for effective immobilization of biomolecules in the microfluidics for biosensing applications. The integrated platform was used toward for the detection of anti-BGH (also called bovine somatotropin or bST) based on Ag-Ab interaction.

The results show that the sensor can reach a very-low detection limit as low as 2 ng/ml (100 pM) in the detection of BGH. In addition, the results show a high response time of 90 sec for the detection of growth hormone. The sensitivity of the system can be improved by further optimizing the platform dimensions, reducing Young’s modulus of the PDMS by using lower ratio PDMS for device fabrication, improving the buried microfluidic system to increase surface area of the platform, and improving the cantilever deflection measurement technique and resolution. The results of this work present a successful demonstration of application of nanocomposite integrated suspended polymeric microfluidics for ultra-high sensitive detection of biomolecular recognition. Indeed, utilization of SPMF compared to silicon-based suspended microfluidics allows us to take rapid strides toward to the next generation of ultra-sensitive biosensors and proves that the detection of biomolecule with concentrations as low as femtomolar (fM) could be possible.

## Electronic supplementary material

Supplementary Information
