# Supplementary material for: Nano-Integrated Suspended Polymeric Microfluidics (SPMF) Platform for Ultra-Sensitive Bio-Molecular Recognition of Bovine Growth Hormones
Source: Sci Rep. 2017 Sep 8;7:10969. doi: 10.1038/s41598-017-11300-2 (PMC5591301; doi:10.1038/s41598-017-11300-2)
Supplement: Supplementary file 1 — Supplementary Information [file 41598_2017_11300_MOESM1_ESM.pdf]

Nano-Integrated Suspended Polymeric Microfluidics (SPMF) Platform for Ultra-Sensitive Bio-Molecular Recognition of Bovine Growth Hormones

Hamid SadAbadi [1,2], Muthukumaran Packirisamy [1,*]

[1] Optical Bio-Micro Systems Laboratory, Department of Mechanical Engineering, Concordia University, EV 4.139, 1455 de Maisonneuve Blvd. W, Montreal, QC, H3G 1M8, Canada
[2] BioMEMS and Bioinspired Microfluidic Laboratory, Department of Mechanical Engineering, University of Calgary, 2500 University Dr. N.W., EEEL 455B, Calgary, AB T2N 1N4 Canada
[*] pmuthu@alcor.concordia.ca

Supplementary Information

Material

Sylgard® 184 elastomer kit from Dow Corning Corporation was used for PDMS fabrication and SU-8-2035 and SU-8-2010 was purchased from MicroChem. For AuNP synthesis, gold chloride trihydrate ($HAuCl_4.3H_2O$) was purchased from Alfa Aesar and 1-pentanol, toluene, N, N-dimethylformamid, 2-propanol and ethyl alcohol, from Sigma-Aldrich. Nanothink® 18, TWEEN® 20, N-Hydroxysuccinimide (NHS), and N-(3-Dimethylaminopropyl)-N′-ethylcarbodiimide hydrochloride (EDC) were purchased from Sigma Aldrich. A monoclonal Bovine Growth Hormone antibody (anti-BGH) in mouse known as bovine somatotropin (bST) and its antigen were purchased from Dr. Parlow National Hormone and Peptide Program in California, USA.

Fabrication of SPMF

For the fabrication of SPMF biosensing platform, SU-8 photoresist patterning technique was used to prepare two molds which were used further to fabricate the two thin layers of the

cantilever. A schematic of a cantilever is shown in Figure 1-a. As it can be seen in this figure, the platform contains 4 layers including two thick substrate layers that hold the cantilever layers and two thin layers that are including: (1) top cover layer to close the channel with the thickness of t_1 and (2) a microfluidic-structure layer containing the buried microchannel with the thickness of t_2. Thick substrate layers are flat PDMS layers that can be fabricated easily by molding PDMS on a flat surface. However, for the fabrication of thin layers two SU-8 molds for cover layer and microfluidic-structure layer were fabricated and explained in detail here.

Preparation of top thin layer is a one-step standard SU-8 process as shown in Figure S1-A. Five molds with different thicknesses (t_1) were fabricated and used to prepare cantilevers in order to find the optimized thickness of the cantilever cover layer of SPMF. SU-8 was spin coated with five different thicknesses of t_1=15 µm, t_1=25 µm, t_1=40 µm, t_1=60 µm, and t_1=105 µm on a cleaned 4" wafer (Figure S1-A, step2) and then soft-baked at 95°C for 3-20 min. The soft-backed SU-8 was exposed to UV light followed by a post-baking process for 1-10 min at 95°C (Figure S1-A, step 3). Afterwards, the SU-8 was developed in SU-8 developer. Finally the mold is hard-baked at 200°C for 15 minutes (step 4). The mold was silanized in vapor phase to enable the removal of PDMS cover layer from the mold after curing.

The procedure for the fabrication of SU-8 mold of µF-structure layer is schematically show in Figure S1-B. It starts with spin coating of SU-8_{2010} to a thickness of h=15µm on a cleaned 4" wafer (Figure S1-B, step 2) and then soft-baking at 95°C for 5 min. The soft-backed SU-8 was exposed to UV light followed by a post-exposure baking (PEB) at 65°C for 5 min as depicted in step 3. The PEB should be performed at a low temperature of 65°C due to two main issues: (i) Even at low-temperature PEB, an image of the pattern would be visible in the SU-8 2010 coated layer. These images can be used as reference to align the second mask. (ii) low-temperature PEB

avoids the appearance of bubbles on the un-exposed parts of SU-8. As a result, low-temperature PEB does not deteriorate the smoothness of first SU-8_{2010} surface which is going to be used for the second SU-8_{2035} spin coating. The second SU-8_{2035} layer was spin coated on the first layer to the thickness of t_2=45 μm followed by soft baking of the wafer at 95°C for 7 min (step 4). Then, the wafer is exposed to UV light followed by PEB at 95°C for 5 min. It should be noted that before the second UV exposure, the mask should be aligned to the previous patterns by using the visible image of the previous layer's features. This is a very important step as any miss-alignment will cause that the buried microchannel will not be at the center of the cantilever. In the next step, the mold is developed in SU-8 developer and the molds are obtained as shown in step 6 of Figure S1-B. Finally the mold is hard-baked at 200°C for 15 minutes. Similarly, the mold was silanized in vapor phase to enable the removal of PDMS from the mold after curing.

Figure 1-b (of main article) shows the fabricated mold for the μF-structure layer of SPMF. In this figure the close-up images show different SU-8 level of patterns. The lower lever will make the buried microchannels. The inset image in this figure shows the mold that was used for the fabrication of cover layer.

After fabrication of SU-8 molds, the PDM was molded for the fabrication of PSMF. PDMS compound was prepared by mixing the base polymer (pre-polymer) and the curing agent (cross-linking agent) with the weight ratio of 10:1 followed by the removal of air bubbles in a vacuum desiccator. Substrate layers are fabricated with the thickness of 3-4 mm by using a simple flat surface as a mold. The layers were then peeled off from the surface and were cut into rectangular shapes. Thin layers of the SPMF were fabricated by using the PDMS thin layer fabrication method. The fabrication process for two thin layers of cover layer and μF-structure layer are shown schematically in Figure S1-C (a and b), respectively.

The fabrication of cover layer and µF-structure layers are similar. First, PDMS was poured into the mold and then a semi-silanized glass slide was placed on PDMS to let the excess of PDMS to be squeezed out of the mold (Figure S1-C, step 1). While keeping the pressure one the glass slide, the PDMS thin layer was cured at 80°C for two hours (step 2). Afterwards the semi-silanized glass slide along with PDMS thin layer can be easily peeled off from the mold as the mold was already fully silanized (step 3). Figure S1-C (c) shows the bonding of two thin layers to form a closed microchannel at the cross-section of cantilever. Figure S1-D shows fabricated thin layers on a semi-salinized glass slide.

After the fabrication of all the four layers, the top substrate was bonded to the cover layer by using oxygen plasma. Then the bonded top substrate carrying cover layer can be removed from the semi-silanized glass. Then the µF-structure layer was bonded to the cover layer and then the whole cantilever was removed from the other semi-silanized glass. Finally, the bottom substrate was bonded (using oxygen plasma) to have the whole PDMS cantilever platform. One important issue in the fabrication of the cantilever is the correct alignment of the cover layer and µF-structure layer before PDMS-PDMS bonding. Any miss-alignment in the bonding process will result in the leakage of the microfluidic system. All the processes should be performed under an optical microscope. Image of a fabricated SPMF is shown in Figure 1-c with inlet and outlet connectors.

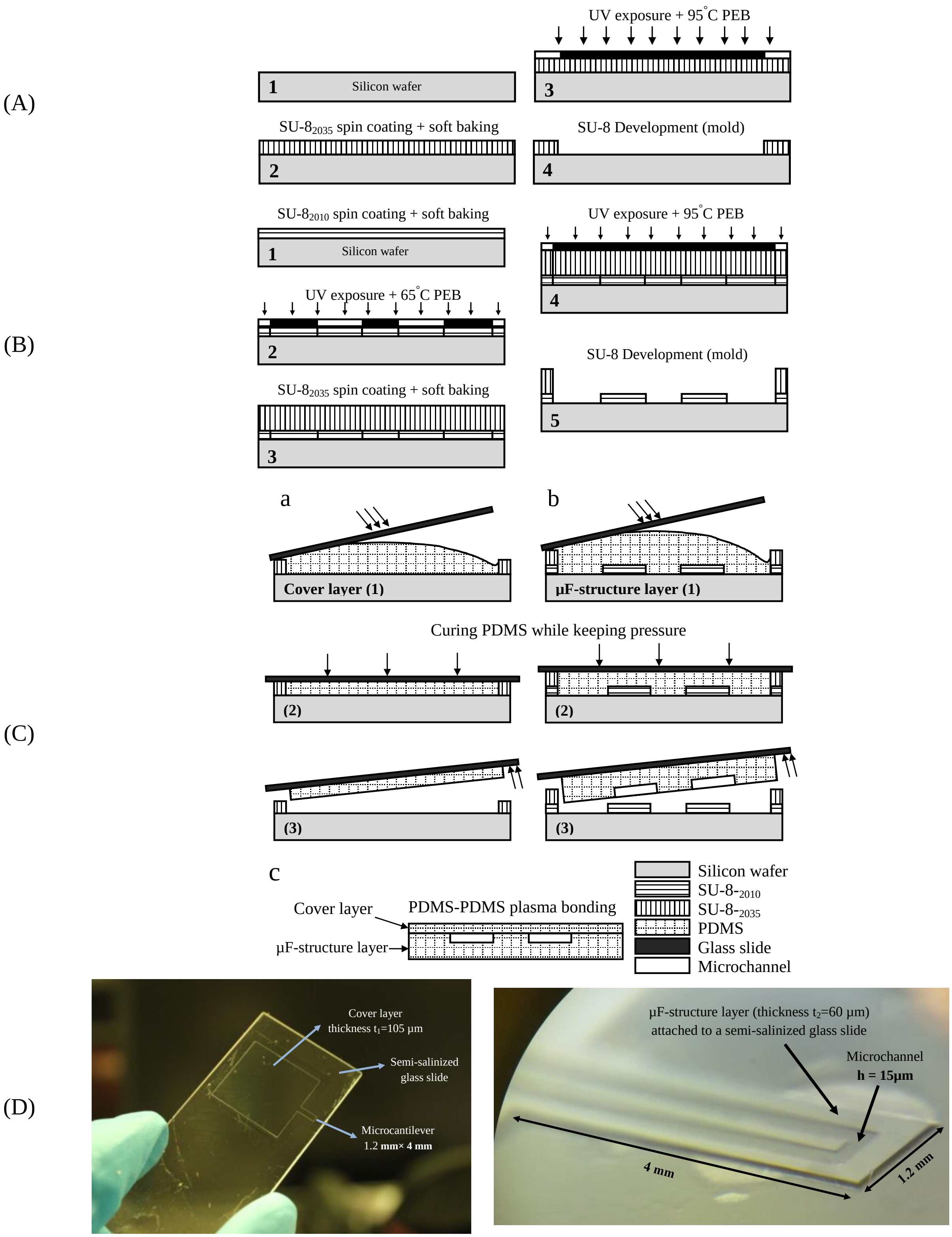

Figure S1: Fabrication of SPMF: (a) fabrication of SU-8 mold for the thin cover layer; (b) fabrication of SU-8 mold for the thin microfluidic-structure layer ; (c) thin PDMS layer fabrication method for making cover layer and µF-structure layer from the SU-8 molds. PDMS-PDMS plasma bonding of thin layers used to form a closed microchannel; (d) Fabricated thin cover layer (left) and µF-structure layer (right) on a semi-salinized glass slide.

In-situ Au-PDMS nanocomposite fabrication

An *in-situ* synthesis method has been previously developed and optimized for microfluidic-based biosensing applications [1]. This method is used to integrate AuNPs onto the surface of PDMS to create a nanocomposite film as the selective layer in the microchannels of fabricated SPMF platform. For synthesis, aqueous solution of chloroauric acid with 2% concentration was prepared and then introduced into the buried microchannel of SPMF by a syringe and then both inlets and outlets are sealed to stop solution evaporation. Then the chip was kept at room temperature for 48hrs. After the incubation time the solution was removed and the channel was washed with DI water. During the course of incubation time, the channel turns to red showing the NP seed initiation and growth of AuNPs inside the microchannel.

The curing agent (cross-linking agent) in PDMS compound reduces the gold ions to gold nanoparticles embedded into the polymer network [1]. A schematic of the process of *in-situ* synthesis reaction at the cross-section of microcantilever and formation of AuNPs is illustrated in Figure 3-a.

The following *in-situ* reduction reaction has been suggested between the gold ions and cross-linking agent of PDMS [2]:

$$3\underset{|}{\overset{|}{Si}}-H+3/2\;H_2O+2AuCl_4^- \rightarrow 3/2-\underset{|}{\overset{|}{Si}}-O-\underset{|}{\overset{|}{Si}}-2Au+8Cl^-+6H^+$$

Upon the introduction of the gold solution, the curing (cross-linking) agent of the PDMS will act as a reductant and initiate the gold nanoparticle to crystalize and grow into the PDMS polymer network as illustrated in Figure 3-a. The *in-situ* reduction reaction ensures high stability of the

AuNPs on the PDMS surface as the particles are embedded into the polymer and interact strongly with the polymer network and thus forming nanocomposite in the SPMF channel. Indeed, our experiments verify that the leaching of particles can be ignored. However, the *in-situ* process is a slow process occurring over 48hrs because of the heterogeneous nature of the reaction. The amount of the cross-linking agent (curing agent) available for the reduction of Au^{3+} is not enough and therefore migration of the cross-linking agent toward the PDMS surface during the reaction is another issue that explains the slow reaction. Possible migration of cross-linking agents of PDMS is schematically shown by dashed arrow in Figure 3-a.

After *in-situ* synthesis, heat treatment was used in order to improve the distribution of AuNPs in PDMS [1,2]. SEM images after the incubation process show that there are many aggregated AuNPs all over the surface. Annealing process has been optimized to reduce the particles aggregation to obtain more uniform distribution of particles on PDMS surface. For annealing process, the fabricated SPMF platform was kept at 300°C for 10 min. SEM images of the AuNPs inside the buried microchannel of the annealed sample showing uniform particle size with average size of 125±5nm as shown in Figure 3-b. The size distribution histograms were extracted from the SEM images using a data visualization modular program. The size distribution histogram of the nanocomposite shows a very narrow size distribution of the particle with less than 8% size variation (125nm±5nm). High uniformity of the synthesized particles makes the composite cantilever platform a suitable candidate for sensitive biosensing experiments.

Biosensing Protocol

Immobilization of Ab on the gold nanoparticles is carried out though functionalizing the nanoparticles with 5mM 11-mercaptoundecanoic acid (NanoThink® ACID11) in ethanol

(5mM), by introducing the solution into the microchannel and keeping it for at least 5 h. Before attaching the Ab to the linkers, which are connected to AuNPs from the previous step, the linkers should be activated. The conventional carbodimide coupling chemistry (EDC/NHS) is used to activate the carboxyl groups. The activation step was carried out by injecting a mixture (with the volume ratio of 1:1) of 0.4M N-(3-dimethylaminopropyl)-N'-ethylcarbodiimide hydrochloride (EDC) and 0.1M N-hydroxysuccinimide (NHS) into the microchannel for 1 hour. The activated linkers are now ready to host Ab. Upon introduction of 200ng/ml solution of antibody in PBS buffer solution for 8 h, Ab is immobilized on the AuNPs. The process is followed by washing the microchannel several times with a TWEEN® 20 solution in PBS (0.05%). The last step in biosensing protocol is the introduction of complementary Ag dissolved in PBS buffer solution. The antigen solution was introduced into the channel by using a syringe pump at a flow rate of 10µl/min. When the Ag solution is injected into the channel, they interact with the immobilized Ab molecules and induce a surface-stress which bends the cantilever. The biosensing protocol has been schematically shown in Figure S2. In this figure, the presence of synthesized AuNPs on the surface of PDMS in the microchannel is shown and through the biosensing protocol, Ab and then Ag have been immobilized on the surface of AuNPs.

After each experiment, the buried microchannel was washed several times with the 0.01% Tween solution and DI water and dried in an oven at 50°C to make sure that there is no more antigen/antibody immobilized on the AuNPs. After the washing process, the cantilever was used for another biosensing experiments.

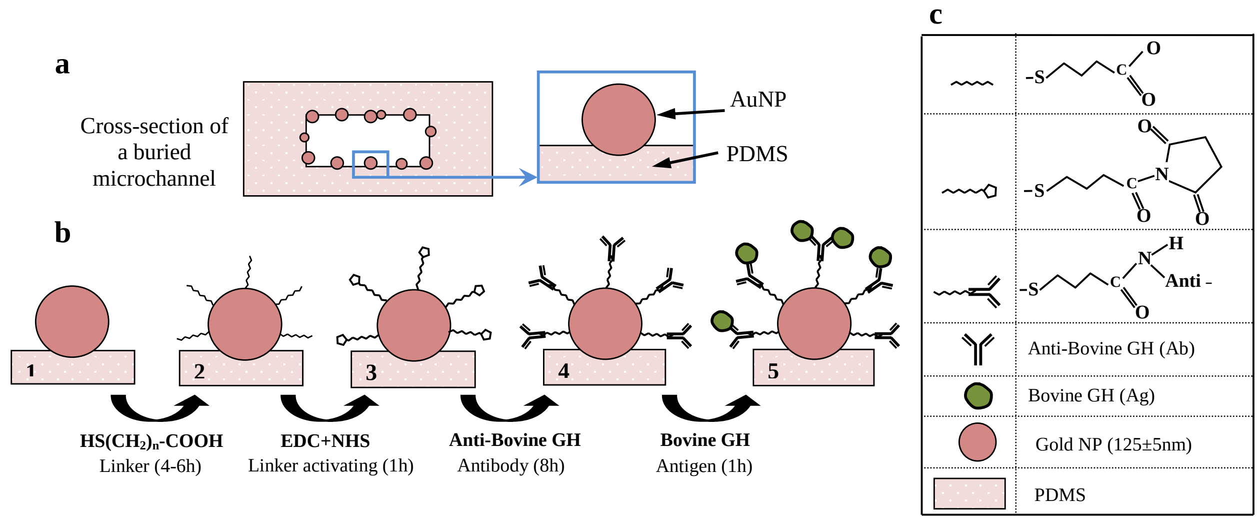

Figure S2: (a) A cross section of the buried microchannel of SPMF and the formation of AuNPs on the PDMS surface; (b) four steps of the biosensing protocol to immobilize Ab of the surface of AuNP; (c) legend of the figure.

Reference

1 SadAbadi, H., Badilescu, S., Packirisamy, M. & Wüthrich, R. Integration of gold nanoparticles in PDMS microfluidics for lab-on-a-chip plasmonic biosensing of growth hormones. *Biosens. Bioelectron.* **44**, 77-84, doi:http://dx.doi.org/10.1016/j.bios.2013.01.016 (2013).

2 SadAbadi, H., Badilescu, S., Packirisamy, M. & Wuthrich, R. PDMS-Gold Nanocomposite Platforms with Enhanced Sensing Properties. *J. Biomed. Nanotechnol.* **8**, 539-549 (2012).
